# Sublethal Effects of Neonicotinoids: How Physiological and Behavioral Disruptions in Non-Target Insects Threaten Biodiversity and Ecosystem Services

**DOI:** 10.3390/insects17010026

**Published:** 2025-12-24

**Authors:** Sarah K. Spence, Shorooq A. M. Alharbi, Afure Ejomah, Feizollah A. Maleki, Michael S. Wolfin, Mônica F. Kersch-Becker

**Affiliations:** 1 Department of Entomology, The Pennsylvania State University, University Park, PA 16802, USA; sks6958@psu.edu (S.K.S.); saa6516@psu.edu (S.A.M.A.); aje5500@psu.edu (A.E.);; 2 Center for Chemical Ecology, Center for Pollinator Research, Center for Insect Biodiversity, One Health Microbiome Center, The Huck Institutes of the Life Sciences, The Pennsylvania State University, University Park, PA 16802, USA

**Keywords:** beneficial arthropods, biological control, ecological services, parasitoids, pesticides, pollinators, predators

## Abstract

Neonicotinoid insecticides were initially hailed as safer alternatives to organochlorine and organophosphate pesticides due to their perceived lower toxicity to non-target organisms. However, it has been recently discovered that sublethal exposure to neonicotinoids negatively affects beneficial arthropods that are essential for a functional ecosystem. These beneficial arthropods include pollinators, biological control agents, and decomposers. This review synthesizes current research on the physiological, behavioral, and reproductive consequences of neonicotinoids on non-target arthropods and their broader ecological impact. The chemical and physical properties of neonicotinoids raise concerns about long-term ecological consequences of neonicotinoid use because these chemicals are persistent in plants and soil, which contributes to prolonged exposure risks for organisms. Sublethal doses of neonicotinoids can disrupt the ecological services provided by these organisms by impairing essential biological processes including motor function, odor detection, development, and reproduction in insects, while also altering behavior such as foraging, mating, and nesting. Furthermore, neonicotinoid exposure can alter community structure, disrupting trophic interactions and food web stability. Recognizing the sublethal impacts of neonicotinoids is critical for the development of more sustainable pest management strategies. It is imperative that future research investigates the underlying mechanisms of sublethal toxicity and identifies safer, more effective approaches to neonicotinoid-based pest control to mitigate adverse ecological effects. Incorporating this knowledge into future environmental risk assessments will be essential for protecting biodiversity and maintaining ecosystem functionality.

## Simple Summary

Neonicotinoids are a type of insecticide that were once considered safer for the environment than other pesticides. But recent research shows that even very small, non-lethal amounts of these chemicals can harm insects that provide ecosystem services—like bees that pollinate crops, insects that eat pests, and species that help break down dead plants and animals. These insecticides linger in soil and plants for long periods, causing changes in how insects move, smell, reproduce, and behave. Over time, this can disrupt entire food webs and weaken important natural processes that keep ecosystems healthy. To protect biodiversity and ensure sustainable farming, it is important to understand how low levels of pesticides can impact beneficial insects. Future research should focus on developing better pest control solutions that do not negatively affect the insects we rely on.

## 1. Introduction

Neonicotinoid insecticides were developed to address concerns about the high toxicity of organochlorine and organophosphate pesticides on non-target organisms, and they became widely used in urban and agricultural systems during the 1990s [1]. Neonicotinoids are now the most widely used class of insecticides globally, accounting for over 20% of the market, due to their systemic movement within plants, extended residual activity, and broad-spectrum insecticidal properties, despite recognized toxicity to non-target organisms [2,3,4,5,6]. The sublethal effects of neonicotinoids on beneficial and non-target organisms have raised increasing concerns despite their efficacy at controlling herbivorous pests [7,8,9,10]. Existing research has largely focused on the lethal effects of neonicotinoids and therefore significant gaps remain in understanding their sublethal impacts on non-target insects.

Neonicotinoids are absorbed and move systemically within plants. This mode of action exposes the targeted herbivorous pests and other organisms throughout the food web [9]. The impacts of insecticides are broadly categorized as lethal or sublethal. Lethal insecticide effects arise from any exposure that causes death in an individual or population, whereas sublethal effects occur from any survivable exposure that alters an organism’s biology, physiology, or behavior [11]. These sublethal effects can contribute to the global decline of insects by profoundly influencing organismal, population, and community dynamics. Global declines in insects disrupt ecosystem processes and reduce overall productivity of cropping systems [12].

**Figure 1:**
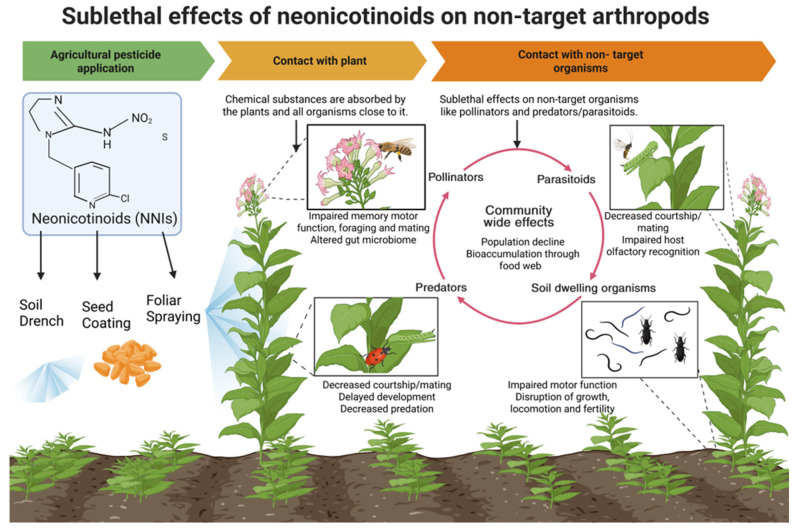
Effects of neonicotinoids though food web in agroecosystem and adjacent habitats. Image created in BioRender [14].

This review synthesizes evidence on the sublethal effects of neonicotinoids on beneficial insects and explores how these impacts propagate to key ecosystem services, including pollination, biological pest control, and biodiversity maintenance, discussing ecological consequences that are often overlooked. Exposure to sublethal doses of neonicotinoids can impair the physiology and alter the behavior of these non-target organisms and disrupt the critical ecological services they provide (Figure 1, Table 1). Beneficial insects such as pollinators and biological control agents provide ecosystem services valued at approximately US $57 billion per year to in the United States [13]. This review first examines the pathways through which neonicotinoids reach non-target arthropods in the environment. It then delves into the sublethal impacts on key physiological and behavioral traits, including development, motor functions, reproduction, olfactory responses, foraging behavior, and social interactions. Finally, we discuss how these sublethal effects cascade through ecological communities, diminishing essential services and processes that support ecosystem stability and productivity. We emphasize the urgent need to better understand the various routes of exposure to neonicotinoids and their impacts on the physiology and ecology of nontarget organisms by synthesizing existing literature and identifying critical research gaps. This knowledge should be incorporated into future environmental risk assessments to protect biodiversity and maintain ecosystem functionality.

## 2. How Do Neonicotinoids Reach Non-Target Organisms

Pesticide exposure routes of non-target organisms, including beneficial predators and pollinators, have changed over time with the adoption of different application strategies. For example, foliar sprays and broadcast treatments have exposed many non-target organisms to high levels of aerial drift. Neonicotinoids applied as seed coatings were developed to increase systemic uptake into plant vascular tissues and reduce airborne exposure for many non-target organisms. However, while these formulations lower the risk of aerial contamination, they significantly increase the likelihood of neonicotinoids leaching into soil and accumulating in terrestrial and aquatic ecosystems [109]. Their physicochemical properties—high water solubility, mobility in soil, and low soil adsorption—facilitate their persistence and movement in the environment [110,111]. It estimated that target plants absorb only a small fraction, 2 to 20%, of the applied neonicotinoids [109]. The remaining 80 to 98% leaches into soil and aquatic ecosystems, where it can persist for up to 8 months [112]. Neonicotinoids exhibit extensive environmental persistence, driven by their ability to propagate through ecosystems, as residues from seed-treated plants can be absorbed by untreated cover crops [113]. Seed coatings and granule formulations reduce aerial exposure but simultaneously facilitate persistent contamination through soil and water, complicating efforts to evaluate their ecological safety [114]. Plant-associated arthropods, like pollinators and omnivorous insects, get exposed to neonicotinoid accumulations in various plant products, including pollen, floral and extra-floral nectar, and guttation droplets [115,116]. Additionally, natural enemies, predators, and parasitoids exposed to nectar and pollen containing neonicotinoids decreased prey consumption, female reproduction, foraging success, mobility, survivorship, and altered sex ratios compared to unexposed controls [57,100,107,117]. Natural enemies are exposed to neonicotinoids not only through feeding on contaminated prey but also by interacting with treated plants, which many carnivorous arthropods use for shelter, supplemental feeding, or while searching for mates [72]. For example, the survival of *Anagyrus pseudococci* parasitoids was reduced after parasitizing the citrus mealybug *Planococcus citri* that fed on neonicotinoid-treated citrus plants compared to control citrus plants [105]. While the high absorbance of neonicotinoids by plants may reduce direct exposure to non-target organisms, it presents a persistent sublethal risk to insects interacting with plants or consuming contaminated prey.

## 3. Physiological Effects

Neonicotinoids share a similar mode of action across all insects. Neonicotinoids exhibit higher potency and selectivity compared to their naturally occurring analog, nicotine [118]. They function by binding to nicotinic acetylcholine receptors (nAChRs), which are agonist-gated ion channels responsible for rapid excitatory neurotransmission in the central nervous system [119]. Binding of neonicotinoids to nAChRs facilitates the accumulation of acetylcholine in the insect nervous system and an influx of calcium ions to the brain [120,121]. At lethal doses, this acetylcholine buildup results in paralysis and death [120,122]. At sublethal doses, however, this buildup affects insect movement, cellular processes, development, and reproduction [15,51,52,83]. Neonicotinoids have the added dimension of time-cumulative toxicity, in which sublethal effects may continue until the repeated doses cause a critical level of neuronal damage or death in the insect [123]. Neonicotinoids significantly impair the motor function of insects exposed to sublethal concentrations, with the mechanisms and severity varying across species [15,22,30,124]. In bees, exposure to neonicotinoid residues disrupted motor functions, reducing flight duration, impairing righting reflexes, inducing repeated circular movements, and increasing grooming behaviors [15,18,22,23,26,125]. Exposed bees also exhibited knockdown, trembling, erratic movements, heightened activity, and tremor following neonicotinoid exposure [16,19,21,28]. These pesticides also impair the movement and foraging ability of the predatory beetles *Harpalus pennsylvanicus*, with these effects persisting for several days post-exposure [30]. Short-term hypermobility occurred in the predatory beetle *Platynus assimilis* and the decomposer burying beetle *Nicrophorus americanus*, and reduced feeding arose in multiple predatory carabid species after exposure [31,32,84].

Reduced movement in insects has primarily been linked to the binding of neonicotinoids to insect nicotinic acetylcholine receptors (nAChRs). Sublethal imidacloprid, clothianidin, and thiamethoxam exposures alter nicotinic acetylcholine receptor (nAChR) subunit expression in bees and other insects, and different receptor subtypes show variable binding affinity [126]. These exposures also modulate the transcription of genes involved in immunity, stress responses, and neural plasticity, including increased expression of the multifunctional gene *vitellogenin* and downregulation of *creb* and *pka*, which are linked to long-term memory [36]. However, the γ-aminobutyric acid (GABA) receptor Rdl (resistant to dieldrin) has been proposed as a secondary target for neonicotinoids, demonstrating the complexity of their neurotoxic effects [127]. Acetylcholine and γ-aminobutyric acid also regulate critical biological processes, many of which are disrupted by neonicotinoids [48,128]. These processes include circadian rhythm regulation, sleep, memory, and olfactory learning in insects [41,47,128,129]. Honeybees *Apis* spp. exhibited decreased olfactory memory and learning ability after larval exposure to neonicotinoids [42,130,131]. Sublethal doses of imidacloprid damaged memory formation in the mushroom bodies, which are key regions of the insect brain involved in short- and medium-term memory [40]. Damselfly larvae *Lestes congener* exposed to neonicotinoids similarly demonstrated a decrease in learned recognition toward predatory fish odor cues [46]. Neonicotinoids’ ability to spontaneously bind to various neurotransmitter receptors in the insect nervous system further exacerbates their detrimental effects, impairing multiple physiological and behavioral processes critical for survival.

Sustained binding of neonicotinoids causes cellular damage from oxidative stress. Neonicotinoid binding to nAChRs opens ion channels but also prevents channel closure by inhibiting hydrolysis by acetylcholinesterase. Calcium ions flood neurons and activate enzymes that increase reactive oxygen species (ROS). Fruit fly *Drosophila melanogaster* larvae exposed to imidacloprid exhibited increased ROS, which lowered ATP production and reduced activity of mitochondrial enzymes [121]. Similarly, aquatic midge *Chironomus dilutus* and honeybee *Apis mellifera* experienced swelling and degeneration of the mitochondrial matrix in imidacloprid-exposed cells [35,39]. Oxidative stress from neonicotinoids resulted in nervous system damage and loss of energy production in both insects. Stress led to downregulation of *creb* and *pka* proteins linked to memory and learning [36,39]. This cellular damage may explain the diminished mobility and impaired learning seen in neonicotinoid-treated arthropods. Increases in anti-apoptotic proteins further demonstrates cellular damage from stress. Heat shock proteins (HSP70), which prevent cell death, were upregulated in adult stingless bee *Melipona scutellaris* after thiamethoxam exposure [37]. Glutathione-S-transferase (GST), a detoxification enzyme, similarly increased in the stingless bee *Scaptotrigona postica* and silk moth *Bombyx mori* after neonicotinoid exposure [38,132].

Neonicotinoids also exhibit a hormetic effect, a phenomenon in which low doses of an environmental agent, such as an insecticide, elicit stimulatory or seemingly beneficial effects [133]. For instance, residual low doses of neonicotinoids enhanced reproduction in the spined soldier bug *Podisus maculiventris* [55] and improved host-finding behaviors in the whitefly parasitoid *Encarsia formosa* [58,134]. In bumblebees, neonicotinoid exposure initially increased the speed of foraging learning but ultimately reduced long-term foraging efficiency [68]. This initial improvement may arise from certain bees’ preference for neonicotinoid-treated sucrose solution, since the bumblebee *Bombus terrestris* consumed more and traveled to neonicotinoid feeders even after feeder relocation [65]. Bumblebees preferred neonicotinoid-treated sucrose in a two-choice bioassay, despite neonicotinoids reducing their overall consumption and the adverse effects on their overall performance and health [9,64]. The hormetic effect raises significant concerns about prolonged exposure, as foraging insects may actively seek out neonicotinoid-treated food sources, inadvertently increasing their risk of sublethal impairments.

Sublethal doses of neonicotinoids significantly impact neural activity in various insect species altering sensory detection and behavior [44,135]. Tatarko et al. [135] investigated the effects of different imidacloprid concentrations on fruit fly *D. melanogaster* behavior and electrophysiological antennal responses. They found that sublethal imidacloprid exposure reduced activity in a single olfactory sensory neuron and delayed the recovery of the antennal response to baseline levels. Moreover, neonicotinoids also can impair other sensory systems. For instance, insecticide exposure reduced visual motion detection and decreased neuronal conduction in axons in the migratory locust *Locusta migratoria* [136]. Similarly, neonicotinoid doses increased honeybee *Apis mellifera* antennal responses to floral odors and queen pheromones, but expedited signal degeneration to floral odors [74]. While electroantennograms provide critical insights into olfactory disruptions caused by neonicotinoids, alternative mechanisms, such as brain dysfunction, may also be involved. Reduced learning performance and responsiveness in bumblebees and honeybees exposed to sublethal doses of neonicotinoids have been linked to reduced brain growth and altered gene expression [17,43]. However, sensory effects can vary among insect species. For example, sublethal doses of imidacloprid impaired both odor and color detection of the pollinating paper wasp *Polistes fuscatus* [45], but only damaged floral volatile odor detection in the bumblebee *Bombus impatiens* with no impact on color detection [44]. This finding highlights the species-specific nature of neonicotinoid impacts on sensory systems and underscores the potential for sublethal exposure to disrupt key ecological processes. For bumblebees, the inability to recognize floral volatiles may hinder efficient foraging, as scent cues are vital for locating nectar and pollen sources. Such impairments could reduce resource intake, reproductive success, and pollination efficiency. Furthermore, this sensory disruption may shift plant–pollinator interactions, favoring plants with more visually prominent cues while disadvantaging those reliant on olfactory signals.

Sublethal neonicotinoid exposure interferes with the development of beneficial insects. Low doses of neonicotinoids delayed the developmental time between larval instars and pupation in the coccinellid seven-spotted lady beetle, *Coccinella septempunctata* [52]. Similar effects have been observed in predatory lacewings *Chrysopa pallens*, bumblebee *Bombus terrestris,* and honeybee *Apis mellifera* larvae, which exhibited prolonged larval and pupal development times following neonicotinoid exposure [49,53,126]. In stingless bee *Scaptotrigona* aff. *depilis*, pupal development was prolonged while larval development was shortened, resulting in asymmetric adults [50]. Even invertebrates without larval stages, such as nematodes *Caenorhabditis elegans*, experienced delayed growth and reduced mobility after exposure to imidacloprid [34].

Recent studies have shown that some insects can detoxify or sequester these neonicotinoids. For example, monarch butterflies can tolerate high concentrations of neonicotinoids because their detoxification pathways have evolved to detoxify cardenolides from milkweed [137]. However, detoxification does not always eliminate the toxic effects, as neonicotinoid metabolites can be just as toxic, or even more so, than the parent compounds [136]. Furthermore, the energy cost of detoxification and sequestration mechanisms could result in physiological costs, leading to long-term population decline despite detoxification.

## 4. Behavioral Effects

As detailed in the previous section, sublethal doses of neonicotinoids affect the central nervous systems of insects, thereby disrupting behaviors critical for survival and reproduction. These disrupted behaviors include foraging, mating, and nesting. The ability of insects to efficiently search for food and shelter is fundamental to their survival. Neonicotinoids targeting herbivorous pests may cause long-term declines in pollinators foraging on treated crops [138]. A growing body of research has investigated how sublethal exposure to neonicotinoids alters nest-founding and foraging behavior in important pollinators [60,66,67,139]. For instance, bumblebee queens exposed to neonicotinoids exhibited delayed nesting behavior, while workers exposed to neonicotinoids were less likely to initiate foraging and nectar feeding [67]. A study in honeybees investigating the effects of two neonicotinoid insecticides, imidacloprid and clothianidin, revealed significant impacts on foraging-trip behavior [61]. While the results varied in a dose-dependent manner, exposure to both insecticides reduced mobility and induced a motionless phase at higher imidacloprid concentrations. Bees displayed abnormal behaviors such as arching their abdomens, flipping upside down, and paddling their legs while lying on their backs at higher concentrations of clothianidin [61]. The functional roles and ecosystem services of pollinators are jeopardized by the significant sublethal effects of neonicotinoids [61].

Sublethal effects of neonicotinoids vary substantially across social insect species. Some colonies exhibited marked behavioral impairments while others appeared relatively unaffected [20,62,63]. Honeybee colonies, for example, often experienced disrupted foraging efficiency and impaired communication due to interference with the waggle dance—a critical behavior used to convey spatial information about food sources. Such disruptions compromise individual foraging success, weaken overall colony cohesion, and reduce the fitness, survival, and reproduction of entire colonies [20,62,63]. Furthermore, stingless bee *Melipona quadrifascitata* displayed fewer social communication behaviors like antennation and trophallaxis after acetamiprid ingestion [71]. In contrast, the southern ant *Monomorium antarcticum* did not display significant sublethal effects on their foraging behavior under comparable neonicotinoid exposure. These divergent outcomes likely stem from fundamental differences in communication and foraging strategies. While social bees integrate both chemical signals and ritualistic behaviors like waggle dancing and trophallaxis, ants rely primarily on pheromone-based chemical communication [76,140]. These ecological and behavioral distinctions may mediate species-specific vulnerability to neonicotinoids. By demonstrating this variability, current findings challenge generalized assumptions about the colony-level effects of neonicotinoids and highlight the importance of species-specific approaches in ecological risk assessment. Evaluating pesticide impacts through a comparative behavioral lens will improve our ability to predict and mitigate risks across diverse taxa of social insects.

Although neonicotinoids did not significantly alter foraging behavior in eusocial ants, these insecticides reduced aggressiveness, thus shifting the outcomes of interspecific interactions such as competition. For instance, *Monomorium antarcticum* exhibited reduced aggressive behaviors—such as biting, spraying acid, and fighting—during encounters with the invasive ants *Linepithema humile* when exposed to imidacloprid. This decline in aggression led to reduced brood numbers and colony size, potentially compromising *M. antarcticum*’s competitive ability against invasive species [76,102]. Similarly, it has been observed that imidacloprid exposure decreased the survival of *Lasius flavus* workers by reducing avoidance behavior and increasing aggression toward co-occurring *Lasius niger* ants [75]. Notably, untreated *L. niger* ants responded with increased aggression toward imidacloprid-treated *L. flavus*, further exacerbating competitive interaction disruptions [75]. These findings suggest that sublethal neonicotinoid exposure can alter interspecific aggression and colony dynamics in social insects, highlighting broader ecological consequences that extend beyond individual-level effects. Sublethal neonicotinoid exposure also reduces the foraging behavior of solitary predaceous arthropods, often resulting in decreased predation rate or prolonged handling times [85,86,88]. For instance, offspring of the seven-spotted lady beetle *Coccinella septempunctata* exhibited reduced aphid prey predation when their parents were treated with thiamethoxam at LC_10_ [79]. Similarly, sublethal doses of thiacloprid reduced the time the hemipteran predator *Macrolophus pygmaeus* spent feeding, while increasing the time it spent resting and preening on treated plants [85]. Similarly, the predaceous beetle *Serangium japonicum* decreased prey attacks and increased prey handling time when foraging on plants treated with thiamethoxam [81]. Adult green lacewing *Chrysoperla sinica* exhibited reduced predation rates when exposed to sublethal doses of imidacloprid [86]. Other predaceous arthropods, such as *Pardosa* and Linyphiidae spiders, experienced both direct and indirect effects of neonicotinoid exposure. The predatory behavior of Linyphiidae spiders was reduced because sublethal topical doses of neonicotinoids resulted in temporary paralysis [88]. In contrast, *Pardosa* spiders killed the prey treated with neonicotinoids but avoided consuming them. This led to a waste of energy spent on hunting without a nutritional payoff [87].

Neonicotinoids can also disrupt the ability of parasitoids to locate and parasitize hosts. Female wasps *Microplitis croceipes* that consumed imidacloprid-treated nectar exhibited reduced ability to locate a host-damaged plant in a wind tunnel, indicating impaired olfactory function [72]. Similarly, in a two-choice olfactometer, female parasitoid wasps *Nasonia vitripennis* treated with sublethal doses of imidacloprid showed a reduced attraction to host pupae compared to the control group, further suggesting that neonicotinoids disrupt insect olfaction [69,70]. In addition, under laboratory conditions, *Psix saccharicola* and *Trissolcus semistriatus* parasitoid wasps displayed lower attack rates and prolonged handling times when parasitizing hemipteran host eggs following exposure to sublethal doses of thiamethoxam and lambda-cyhalothrin [77,78].

Essential ecosystem services, including pollination, biological control, and nutrient cycling, depend on complex behavioral processes that mediate interactions among organisms [13]. Many of these behaviors, such as foraging, host or prey location, and plant-pollinator communication, are chemically mediated and rely on accurate stimulus detection. Sublethal neonicotinoid exposure disrupts these chemically mediated behaviors in both social and solitary insects, impairing foraging efficiency, host-seeking, and sensory processing [67,72,73,81]. Such behavioral changes can cascade through the ecosystem, reducing predation rates, altering predator–prey dynamics, and interfering with pollinator efficiency. Neonicotinoids may further exacerbate these impacts by impairing essential colony-level behaviors, including communication, aggression, and escape responses in social insects [61,141]. Although responses are often species-specific [20,27,76], the consistent pattern of behavioral impairment across functional groups underscores the broad ecological risks posed by neonicotinoids. Understanding and mitigating these sublethal effects is therefore critical for preserving biodiversity and maintaining ecosystem resilience, especially considering the central role of behavior in regulating ecosystem processes [142,143].

## 5. Reproductive Effects

Mating and reproductive output drive population growth [70,97]. Sublethal neurotoxic effects of neonicotinoids can alter key reproductive processes, including mate-searching behavior, detecting mating pheromones, and courtship behaviors [69,70,91]. These disruptions impair individuals’ ability to locate and respond to potential mates, thereby accelerating population declines. In social insects, whose colony success depends heavily on reproductive health, these effects are especially severe [96,97]. Sublethal neonicotinoid exposure compromised honeybee colony health because exposed *Apis mellifera* queens had reduced sperm storage, enlarged ovaries, and decreased egg-laying rates [90]. Additionally, the genetic diversity and resilience of the colony was reduced because exposed queens mated less frequently [89]. Reproductive success in annual social bees, such as the bumblebee *Bombus terrestris*, depends on the production of new queens and males. Colonies exposed to imidacloprid showed an 85% reduction in queen production [95]. Sulfoxaflor exposure similarly reduced the number of reproductive workers and queens [49]. Sublethal doses of thiamethoxam impaired male fertility, resulting in 50% fewer viable sperm in mated queens compared to controls [93]. Neonicotinoid-induced neurological impairments may also reduce feeding efficiency, further constraining reproductive output [96]. These effects are not limited to bees. Sublethal neonicotinoid exposure in ants reduced queen fecundity, colony survival, and worker function [144,145,146]. Neonicotinoids undermine the sustainability of insect populations by interfering with reproductive systems, altering reproductive caste production, and reducing offspring viability. Together, these findings demonstrate that sublethal neonicotinoid exposure undermines insect population sustainability by disrupting reproductive systems, altering reproductive caste dynamics, and reducing offspring viability.

Neonicotinoids can also negatively impact insect reproduction by interfering with detection of and response to sex pheromones, preventing mate-finding and hindering courtship behaviors. Male *Nasonia vitripennis* parasitoid wasps exposed to sublethal doses of imidacloprid exhibited reduced courtship behaviors such as head-nodding and mounting. Additionally, response rates to male-produced sex pheromones were reduced in exposed females compared to control females [69,70]. Mating success declined more sharply in imidacloprid-treated males compared to treated females. Mating success plummeted by 80% when both sexes were exposed [69,70]. Male parasitoid wasps *Spalangia endius* displayed consistent courtship behaviors towards both exposed and unexposed females, but unexposed control females primarily mated with males, and exposed females were unreceptive. In contrast, females presented with exposed males and unexposed males were unlikely experience any courtship behavior from the exposed male, suggesting sex-specific responses to neonicotinoids [91]. Predators also displayed varied responses to sublethal neonicotinoid exposure. Exposure to low doses of neonicotinoids did not impact oviposition, fertility, or survival in the soldier bug *Podisus maculiventris* [55]. The Asian lady beetle, *Harmonia axyridis*, experienced more severe consequences, including negative transgenerational effects in reproduction following larval exposure [83]. In invertebrates, the fertility of *Caenorhabditis elegans* nematodes was reduced following sublethal exposure to neonicotinoids [34]. Courtship and mating behaviors of exposed male *Pardosa* spiders were decreased compared to control spiders [92]. In summary, neonicotinoids may impair reproduction success through various mechanisms, including disrupting pheromone communication, damaging sexual organs, or altering mating behaviors. These disruptions can lower reproductive output, alter the balance of sexual castes, and ultimately suppress population growth, with far-reaching consequences for insect biodiversity and conservation.

## 6. Community-Wide Effects

The widespread use of neonicotinoids has been suggested as a major driver of the global insect decline [147]. Given that insects comprise approximately 50% of biodiversity within ecological communities [148,149], their decline disrupts ecological interactions and destabilizes ecosystems [150]. A meta-analysis of 44 field and laboratory studies concluded that neonicotinoids negatively impacted multiple functional groups of non-target arthropods, including pollinators, predators, parasitoids, omnivores and detritivores [8]. These widespread effects extended beyond direct toxicity, disrupting species interactions and triggering cascading effects throughout food webs [151]. This impact was not only restricted to agroecosystems but was also observed in organic farms and natural habitats adjacent to neonicotinoids application sites [152]. Reduced prey availability limits food resources for higher trophic levels. Consequently, prey populations are released from top-down control, weakening biological control [151].

Neonicotinoids move through trophic levels and bioaccumulate in food webs despite being water-soluble and typically excreted by animals. For instance, accumulation of neonicotinoids in earthworms and slugs paralyzed predatory arthropods such as ground beetles [108,153]. Whiteflies feeding on neonicotinoid-treated tomato plants excreted unmetabolized residues in their honeydew, inadvertently creating a secondary contamination pathway that can expose beneficial arthropods and other honeydew-feeding organisms [106]. Sublethal neonicotinoid exposures altered community composition of soil-dwelling insect communities [29,99,102]. Additionally, neonicotinoid contamination was linked to declines in aquatic insect biodiversity and ecosystem function [154,155,156,157]. For example, increased neonicotinoid concentrations significantly reduced the abundance and biomass of key aquatic insect orders including Coleoptera, Diptera, Ephemeroptera, Odonata, and Trichoptera [158]. These findings demonstrate that neonicotinoids disrupt multiple ecological communities by altering trophic interactions.

These studies collectively highlight the far-reaching consequences of neonicotinoid exposure across multiple ecosystems. Although several studies have documented the direct toxic effects on individual species, the broader ecological disruptions, such as altered species interactions and cascading effects through food webs, remain understudied. In particular, the long-term implications for ecosystem services, stability, and functioning remain poorly understood. Ongoing biodiversity loss across trophic levels threatens to trigger profound and potentially irreversible ecological consequences.

## 7. Concluding Remarks

We examined the empirical evidence on the sublethal effects of neonicotinoids on the physiology, behavior, reproduction, and ecology of non-target arthropods in this review (Table 1, Table S1). The chemical properties of neonicotinoids contribute to their persistence in plants and soil, extending the exposure risk for non-target organisms. These sublethal effects can drive chronic declines of beneficial insects, leading to secondary pest outbreaks and biodiversity loss [158,159]. Declines in pollinators and natural enemies can also result in significant economic and ecological losses. Further research is needed to understand how neonicotinoids affect plant physiology, particularly plant defenses [160,161], as these changes can alter plant–insect interactions and propagate throughout the food web [151]. Importantly, major gaps remain in our understanding of multiple routes through which non-target arthropods are exposed to neonicotinoids, including contaminated nectar and pollen, guttation droplets, soil and leaf litter contact, prey-mediated transfer, and trophic cascades. These pathways determine not only the magnitude of exposure but also the likelihood that sublethal effects accumulate across life stages and trophic levels. Advancing mechanistic knowledge of both exposure routes and arthropod responses will be critical for predicting how sublethal effects spread through ecosystems. Despite mounting evidence of these impacts, long-term consequences of chronic, low-dose exposure on ecological interactions and ecosystems functioning remain poorly understood and should be prioritized in future research. Identifying the mechanisms of sublethal toxicity is crucial for guiding the development of safer insecticides and revising regulatory policies to mitigate their ecological side effects.

The ecological impact of neonicotinoids is often compounded by other stressors, including climate change and pathogen outbreaks. These combined pressures underscore the urgency of understanding how neonicotinoids contribute to biodiversity loss and ecosystem instability. Research on community-level responses remains limited, hampering our ability to predict disruptions to ecological networks and essential ecosystem services. Future studies should integrate long-term field data and experimental approaches to evaluate cascading effects across trophic levels. Advancing our understanding of these dynamics is critical for designing sustainable pest management strategies that reduce unintended environmental harm.

## Figures and Tables

**Table 1:** Summary of the sublethal effects of neonicotinoids on non-target arthropod physiology, reproduction, behavior, and community dynamics. Detailed information on tested dosages is provided in Table S1.

Effect	Organism	Reference
Physiological Effects		
Impaired motor function	*Apis mellifera*	Williamson et al., 2014 [15], Colin et al., 2004 [16], Christen et al., 2021 [17], Hesselbach and Scheiner 2019 [18], Lambin et al., 2001 [19], Medrzycki et al., 2003 [20], Suchail et al., 2001 [21], Tosi and Nieh 2017 [22]
	*Bombus terrestris*	Kenna et al., 2019 [23], Sargent et al., 2021 [24]
	*Bombus impatiens*	Crall et al., 2018 [25]
	*Partamona helleri*	Motta et al., 2024 [26]
	*Protopolybia exigua*	Crispim et al., 2023 [27]
	*Tetragonisca angustula*	Jacob et al., 2019 [28]
	*Tetramorium caespitum*	Penn & Dale 2017 [29]
	*Harpalus pennsylvanicus*	Kunkel et al., 2001 [30]
	*Nicrophorus americanus*	Cavallaro et al., 2025 [31]
	*Platynus assimilis*	Tooming et al., 2017 [32]
	*Deleatidium* spp.	Hunn et al., 2019 [33]
	*Caenorhabditis elegans*	Bradford et al., 2020 [34]
Impaired cellular processes	*Apis mellifera*	Catae et al., 2018 [35], Christen et al., 2016 [36]
	*Melipona scutellaris*	Miotelo et al., 2025 [37]
	*Scaptorigona postica*	Maloni et al., 2025 [38]
	*Chironomus dilutus*	Wei et al., 2020 [39]
Impaired learning or memory	*Apis mellifera*	Decourtye et al., 2004 [40], Piiroinen & Goulson 2016 [41]
	*Apis cerana*	Tan et al., 2015 [42]
	*Bombus terrestris*	Smith et al., 2020 [43]
	*Bombus impatiens*	Muth et al., 2019 [44]
	*Polistes fuscatus*	Corcoran & Tibbetts 2023 [45]
	*Lestes congener*	Wickramasingha et al., 2024 [46]
Impaired sleep or circadian rhythm	*Apis mellifera*	Tackenberg et al., 2020 [47]
	*Bombus terrestris*	Tasman et al., 2020 [48]
Delayed development	*Bombus terrestris*	Siviter et al., 2020 [49]
	*Scaptorigona* aff. *depilis*	Rosa et al., 2016 [50]
	*Coccinella septempunctata*	Jiang et al., 2018 [51]
		You et al., 2022 [52]
	*Chrysopa pallens*	Su et al., 2022 [53]
Hormesis: Stimulation of reproduction	*Trichogramma chilonis* Ishii	Ray et al., 2022 [54]
	*Podisus maculiventris*	Rix and Cutler 2020 [55]
Hormesis: Increased predation and host finding	*Trichogramma chilonis* Ishii	Ray et al., 2023 [56]
	*Tiphia vernalis*	Oliver et al., 2005 [57]
	*Encarsia formosa*	Wang et al., 2019 [58]
Altered gut microbiome	*Apis mellifera*	Alberoni et al., 2021 [59]
Impaired foraging	*Apis mellifera*	Morfin et al., 2019 [60], Schneider et al., 2012 [61], Tison et al., 2020 [62], Tison et al., 2016 [63]
	*Bombus terrestris*	Kessler et al., 2015 [64], Arce et al., 2018 [65]
	*Bombus impatiens*	Leza et al., 2018 [66], Muth & Leonard 2019 [67], Stanley & Raine 2016 [68]
	*Nasonia vitripennis*	Schöfer et al., 2023 [69], Tappert et al., 2017 [70]
	*Melipona quadrifasciata*	Boff et al., 2018 [71]
	*Microplitis croceipes*	Stapel et al., 2000 [72]
Olfactory recognition	*Apis mellifera*	Palmer et al., 2013 [73]
		Favaro et al., 2022 [74]
	*Apis cerana*	Tan et al., 2015 [42]
	*Nasonia vitripennis*	Schöfer et al., 2023 [69]
Increased aggression	*Lasius flavus*	Thiel & Kohler. 2016 [75]
Decreased aggression	*Monomorium antarcticum*	Barbieri et al., 2013 [76]
Decreased predation	*Psix saccharicola*	Ranjbar, Reitz, Jalali, et al., 2021 [77], Ranjbar, Reitz, Sardary, et al., 2021 [78]
	*Trissolcus semistriatus*	Ranjbar, Reitz, Jalali, et al., 2021 [77], Ranjbar, Reitz, Sardary, et al., 2021 [78]
	*Tiphia vernalis*	Oliver et al., 2005 [57]
	*Coccinella septempunctata*	Jiang et al., 2019 [79]
	*Cycloneda sanguinea*	Fernandes et al., 2016 [80]
	*Chauliognathus flavipes*	Fernandes et al., 2016 [80]
	*Serangium japonicum*	Yao et al., 2015 [81], He et al., 2012 [82]
	*Platynus assimilis*	Tooming et al., 2017 [32]
	*Harmonia axyridis*	Zhang et al., 2023 [83]
	*Carabidae* spp.	Pearsons & Tooker 2025 [84]
	*Orius insidiosus*	Fernandes et al., 2016 [80]
	*Macrolophus pygmaeus*	Martinou et al., 2014 [85]
	*Chrysoperla sinica*	Shan et al., 2020 [86]
	*Pardosa agrestis*	Korenko et al., 2019 [87]
	*Pardosa lugubris*	Řezáč et al., 2019 [88]
	*Philodromus cespitum*	Řezáč et al., 2019 [88]
**Reproductive Effects**		
Impaired courtship and mating	*Apis mellifera*	Forfert et al., 2017 [89], Williams et al., 2015 [90]
	*Spalangia endius*	Kremer & King 2019 [91]
	*Nasonia vitripennis*	Schöfer et al., 2023 [69], Tappert et al., 2017 [70]
	*Pardosa agrestis*	Korenko et al., 2020 [92]
Decreased sperm viability	*Apis mellifera*	Williams et al., 2015 [90]
	*Bombus terrestris*	Straub et al., 2022 [93]
	*Osmia cornuta*	Strobl et al., 2021 [94]
Decreased fecundity	*Bombus terrestris*	Whitehorn et al., 2012 [95], Laycock et al., 2012 [96], Baron et al., 2017 [97], Siviter et al., 2018 [98]
	*Bombus impatiens*	Leza et al., 2018 [66]
		Crall et al., 2018 [25]
	*Eucera pruinosa*	Willis Chan and Raine 2021 [99]
	*Nasonia vitripennis*	Whitehorn et al., 2015 [100]
	*Aphidius flaviventris*	Majidpour et al., 2022 [101]
	*Lasius niger*	Schläppi et al., 2020 [102]
	*Chrysoperla carnea*	Gontijo et al., 2014 [103]
	*Coccinella septempunctata*	Jiang et al., 2018 [51]
	*Caenorhabditis elegans*	Bradford et al., 2020 [34]
Reduced egg viability	*Coccinella septempunctata*	Jiang et al., 2019 [79]
	*Eriopis connexa*	Fogel et al., 2013 [104]
	*Harmonia axyridis*	Zhang et al., 2023 [83]
**Community Effects**		
Beneficial insect exposure through parasitism and predation	*Anagyrus pseudococci*	Calvo-Agudo et al., 2019 [105]
		Quesada & Scharf 2023 [106]
	*Aphytis melinus*	Grafton-Cardwell et al., 2008 [107]
	*Comperiella bifasciata*	Grafton-Cardwell et al., 2008 [107]
	*Chlaenius tricolor*	Douglas et al., 2015 [108]

## Data Availability

No new data were created or analyzed in this study. Data sharing is not applicable to this article.

## Supplementary Materials

The following supporting information can be downloaded at: https://www.mdpi.com/article/10.3390/insects17010026/s1, Table S1: Compilation of studies reporting sublethal effects of neonicotinoids on insect physiology, behavior, reproduction, and community interactions, with associated insect taxa, neonicotinoid compounds, and exposure doses.
