# Supplementary material for: Sublethal Effects of Neonicotinoids: How Physiological and Behavioral Disruptions in Non-Target Insects Threaten Biodiversity and Ecosystem Services
Source: Insects. 2025 Dec 24;17(1):26. doi: 10.3390/insects17010026 (PMC12842587; doi:10.3390/insects17010026)
Supplement: Supplementary file 1 [file insects-17-00026-s001.zip › insects-4010745-supplementary.pdf]

Table S1. Compilation of studies reporting sublethal eff

Effect	Order
**Physiological Effects**	
Impaired motor function	Hymenoptera
	Coleoptera
	Ephemeroptera
	Rhabditida (Phylum: Nematoda)
Impaired cellular processes	Hymenoptera
	Diptera
Impaired learning or memory	Hymenoptera
	Odonata
Impaired sleep or circadian rhythm	Hymenoptera
Delayed development	Hymenoptera

	Coleoptera
	Neuroptera
Hormesis: Stimulation of reproduction	Hymenoptera<br>Hemiptera
Hormesis: Increased predation and host finding	Hymenoptera
Altered gut microbiome	Hymenoptera
**Behavioral Effects**	
Impaired foraging	Hymenoptera
Olfactory recognition	Hymenoptera
Increased aggression	Hymenoptera
Decreased aggression	Hymenoptera
Decreased predation	Hymenoptera
	Coleoptera

	Hemiptera
	Neuroptera
	Araneae
**Reproductive Effects**	
Impaired courtship and mating	Hymenoptera
	Araneae
Decreased sperm viability	Hymenoptera
Decreased fecundity	Hymenoptera
	Neuroptera
	Coleoptera<br>Rhabditida (Phylum: Nematoda)
Reduced egg viability	Coleoptera
**Community Effects**	
Beneficial insect exposure through parasitism and predation	Hymenoptera

	Coleoptera

ects of neonicotinoids on insect physiology, be

Organism	Reference	Ref#
Apis mellifera	Williamson et al. 2014	[15]
	Colin et al. 2004	[16]
	Christen et al. 2021	[17]
	Hesselbach and Scheiner 2019	[18]
	Lambin et al. 2001	[19]
	Medrzycki et al. 2003	[20]
	Suchail et al.2001	[21]
	Tosi and Nieh 2017	[22]
Bombus terrestris	Kenna et al. 2019	[23]
	Sargent et al. 2021	[24]
Bombus impatiens	Crall et al. 2018	[25]
Partamona helleri	Motta et al. 2024	[26]
Protopolybia exigua	Crispim et al. 2023	[27]
Tetragonisca angustula	Jacob et al. 2019	[28]
Tetramorium caespitum	Penn & Dale 2017	[29]
Harpalus pennsylvanicus	Kunkel et al. 2001	[30]
Nicrophorus americanus	Cavallaro et al. 2025	[31]
Platynus assimilis	Tooming et al. 2017	[32]
Deleatidium spp.	Hunn et al. 2019	[33]
Caenorhabditis elegans	Bradford et al. 2020	[34]
Apis mellifera	Catae et al. 2017	[35]
	Christen et al. 2016	[36]
Melipona scutellaris	Miotelo et al. 2025	[37]
Scaptorigona postica	Maloni et al. 2025	[38]
Chironomus dilitus	Wei et al. 2020	[39]
Apis mellifera	Decourtye et al. 2004	[40]
	Piiroinen & Goulson 2016	[41]
Apis cerana	Tan et al. 2015	[42]
	Tan et al. 2015	[42]
Bombus terrestris	Smith et al. 2020	[43]
Bombus impatiens	Muth et al. 2019	[44]
Polistes fuscatus	Corcoran & Tibbetts 2023	[45]
Lestes congener	Wickramasingha et al. 2024	[46]
Apis mellifera	Tackenberg et al. 2020	[47]
Bombus terrestris	Tasman et al. 2020	[48]
Bombus terrestris	Siviter et al. 2020	[49]

Scaptorigona aff. *depilis*	Rosa et al. 2016	[50]
Coccinella septempunctata	Jiang et al. 2018	[51]
	You et al. 2022	[52]
Chrysopa pallens	Su et al. 2022	[53]
Trichogramma chilonis Ishii	Ray et al. 2022	[54]
Podisus maculiventris	Rix and Cutler 2020	[55]
Trichogramma chilonis Ishii	Ray et al. 2023	[56]
Tiphia vernalis	Oliver et al. 2005	[57]
Encarsia formosa	Wang et al. 2019	[58]
Apis mellifera	Alberoni et al. 2021	[59]

Apis mellifera	Morfin et al. 2019	[60]
	Schneider et al. 2012	[61]
	Tison et al. 2020	[62]
	Tison et al. 2016	[63]
Bombus terrestris	Kessler et al. 2015	[64]
	Arce et al. 2018	[65]
Bombus impatiens	Leza et al. 2018	[66]
	Muth & Leonard 2019	[67]
	Stanley & Raine 2016	[68]
Nasonia vitripennis	Schöfer et al. 2023	[69]
	Tappert et al. 2017	[70]
Melipona quadrifasciata	Boff et al. 2018	[71]
Microplitis croceipes	Stapel et al. 2000	[72]
Apis mellifera	Palmer et al. 2013	[73]
	Favaro et al. 2022	[74]
Apis cerana	Tan et al. 2015	[42]
	Tan et al. 2015	[42]
Nasonia vitripennis	Schöfer et al. 2023	[69]
Lasius flavus	Thiel & Kohler. 2016	[75]
Monomorium antarcticum	Barbieri et al. 2013	[76]
Psix saccharicola	Ranjbar, Reitz, Jalali, et al. 2021	[77]
	Ranjbar, Reitz, Sardary, et al. 2021	[78]
Trissolcus semistriatus	Ranjbar, Reitz, Jalali, et al. 2021	[77]
	Ranjbar, Reitz, Sardary, et al. 2021	[78]
Tiphia vernalis	Oliver et al. 2005	[57]
Coccinella septempunctata	Jiang et al. 2019	[79]
Cycloneda sanguinea	Fernandes et al. 2016	[80]
Chauliognathus flavipes	Fernandes et al. 2016	[80]
Serangium japonicum	Yao et al. 2015	[81]

	He et al. 2012	[82]
Platynus assimilis	Tooming et al. 2017	[32]
Harmonia axyridis	Zhang et al. 2023	[83]
Carabidae spp.	Pearsons & Tooker 2025	[84]
Orius insidiosus	Fernandes et al. 2016	[80]
Macrolophus pygmaeus	Martinou et al. 2014	[85]
Chrysoperla sinica	Shan et al. 2020	[86]
Pardosa agrestis	Korenko et al. 2019	[87]
Pardosa lugubris	Řezáč et al. 2019	[88]
Philodromus cespitum	Řezáč et al. 2019	[88]
Apis mellifera	Forfert et al. 2017	[89]
	Williams et al. 2015	[90]
Spalangia endius	Kremer & King 2019	[91]
Nasonia vitripennis	Schöfer et al. 2023	[69]
	Tappert et al. 2017	[70]
Pardosa agrestis	Korenko et al. 2020	[92]
Apis mellifera	Williams et al. 2015	[91]
Bombus terrestris	Straub et al. 2022	[93]
Osmia cornuta	Strobl et al. 2021	[94]
Bombus terrestris	Whitehorn et al. 2012	[95]
	Laycock et al 2012	[96]
	Baron et al. 2017	[97]
	Siviter et al. 2018	[98]
Bombus impatiens	Leza et al. 2018	[66]
	Crall et al. 2018	[25]
Eucera pruinosa	Willis Chan and Raine 2021	[99]
Nasonia vitripennis	Whitehorn et al. 2015	[100]
Aphidius flaviventris	Majidpour et al. 2022	[101]
Lasius niger	Schläppi et al. 2020	[102]
Chrysoperla carnea	Gontijo et al. 2014	[103]
Coccinella septempunctata	Jiang et al. 2018	[51]
Caenorhabditis elegans	Bradford et al. 2020	[34]
Coccinella septempunctata	Jiang et al. 2019	[79]
Eriopis connexa	Fogel et al. 2013	[104]
Harmonia axyridis	Zhang et al. 2023	[83]
Anagyrus pseudococci	Calvo-Agudo et al. 2019	[105]
	Quesada & Scharf 2023	[106]
Aphytis melinus	Grafton-Cardwell et al. 2008	[107]

Comperiella bifasciata	Grafton-Cardwell et al. 2008	[107]
Chlaenius tricolor	Douglas et al. 2015	[108]

havior, reproduction, and community in

**Neonicotinoid**
imidacloprid, dinotefuran, thiamethoxam, clothiadin
imidacloprid
thiamethoxam, thiacloprid
flupyradifurone, imidacloprid
imidacloprid
imidacloprid
imidacloprid and metabolites
thiamethoxam
imidacloprid
imidacloprid
imidacloprid
thiamethoxam
thiamethoxam
acetamiprid, imidacloprid, thiacloprid, thiamethoxam
imidacloprid
imidacloprid
imidacloprid
thiamethoxam
imidacloprid
imidacloprid
imidacloprid
acetamiprid,clothianidin, imidacloprid, thiamethoxam
thiamethoxam
thiamethoxam
imidacloprid
imidacloprid
clothianidin
imidacloprid
imidacloprid
imidacloprid
imidacloprid
imidacloprid, sulfoxaflor
imidacloprid
clothianidin, thiamethoxam
imidacloprid
sulfoxaflor

thiamethoxam
clothianidin
acetamiprid
acetamiprid, dinotefuran
imidacloprid
imidacloprid
imidacloprid
imidacloprid, thiamethoxam
acetamiprid, imidacloprid, nitenpyram, thiamethoxam, sulfoxaflor
imidacloprid, thiacloprid

clothianidin
imidacloprid, clothianidin
clothianidin
thiacloprid
imidacloprid, thiamethoxam
thiamethoxam
imidacloprid
imidacloprid
thiamethoxam
acetamiprid, flupyradifurone, sulfoxaflor
imidacloprid
acetamiprid with pyrethroid
imidacloprid
imidacloprid, imidacloprid-olefin, thiamethoxam
imidacloprid, thiacloprid
imidacloprid
imidacloprid
acetamiprid, flupyradifurone, sulfoxaflor
imidacloprid
imidacloprid
thiamethoxam with lambda-cyhalothrin
thiamethoxam with lambda-cyhalothrin
thiamethoxam with lambda-cyhalothrin
thiamethoxam with lambda-cyhalothrin
imidacloprid, thiamethoxam
clothianidin
imidacloprid, thiamethoxam
imidacloprid, thiamethoxam
imidacloprid

imidacloprid
thiamethoxam
acetamiprid
clothianidin, imidacloprid, thiamethoxam
imidacloprid, thiamethoxam
thiacloprid
imidacloprid
acetamiprid, imidacloprid, thiacloprid, thiamethoxam
imidacloprid
imidacloprid, acetamiprid, thiamethoxam, thiacloprid

thiamethoxam, clothianidin
thiamethoxam, clothianidin
imidacloprid
acetamiprid, flupyradifurone, sulfoxaflor
imidacloprid
acetamiprid, thiacloprid
thiamethoxam, clothianidin
thiamethoxam
thiamethoxam
imidacloprid
imidacloprid
thiamethoxam
sulfoxaflor
imidacloprid
imidacloprid
imidacloprid
imidacloprid
thiacloprid with deltamethrin
thiamethoxam
thiamethoxam
thiamethoxam
imidacloprid
clothianidin
acetamiprid
acetamiprid

thiamethoxam, imidacloprid
imidacloprid
imidacloprid

imidacloprid
thiamethoxam

teractions, with associated insect taxa, neonicotinoid compo

Dosage
10nM and 100nM in sucrose solutions *ad libitum*
6ug/kg in sucrose solution *ad libitum*
1.5 ng thiamethoxam/bee and 1.25 ng thiacloprid/bee in sucrose solution *ad libitum*
5 µL of 8.3 umol/L and 83 umol/L, 0.031umol/L and 0.31 umol/L in sucrose solution
1 µL of 2.5, 5, 10, and 20 ng/bee
100 ppb and 500 ppb in sucrose solution *ad libitum*
0.1, 1, or 10 µg/L neonicotinoid in sucrose solution *ad libitum*
1.34 ng/bee acute and 1.42-3.48 ng/bee/day chronic range in sucrose solution
10 ppb imidacloprid in sucrose solution
10 ppb imidacloprid in sucrose solution
0.1 or 1 ng imidacloprid/bee
0.09 ng thiamethoxam/g in sucrose solution *ad libitum*
0.5 µL of 1/10 and 1/100 of 0.0428 ng thiamethoxam/L sucrose solution
173.6 ng acetamiprid, 1.70 ng imidacloprid, 54.09 ng thiacloprid, 0.28 ng thiamethoxam/L in sucrose solu
three seeds treated with 1.2mL imidacloprid/kg seed
0.336 kg imidacloprid/ha sprayed on turfgrass plots and food
58.9 ng imidacloprid/beetle
1081.1, 324.3, 108.1, 32.4, 10.8, and 1.1 ng thiamethoxam/g body weight in 20 uL food mixture
0.9 and 2.1 ug/L in artificial soft water environment
Undiluted pesticide containing imidacloprid applied to plates
0.014651 g imidacloprid/µL diet in sucrose solution *ad libitum*
80-8000 ng acetamiprid, 0.3-30 ng clothianidin, 3-300 ng imidacloprid, and 0.1-10 ng thiamethoxam/µL i
0.000543 ng thiamethoxam/µL diet in sucrose solution *ad libitum*
0.011 ng or 0.0011 ng thiamethoxam/µL in sucrose solution *ad libitum*
0.01-80 ng imidacloprid/µL in hard water
0.5 uL of 25 mg/L or 250 µg/L imidacloprid in sucrose solution
4 ppb clothianidin in sucrose solution *ad libitum*
2 µL/larva/day of 17.7 ppb imidacloprid in sucrose solution
10 µL of 8.9 or 88.7 ppb imidacloprid in sucrose solution
5 ppb imidacloprid in sucrose solution *ad libitum*
20 uL of 22.5 ppb imidacloprid in sucrose solution
0.5 or 2.0 ng/wasp imidacloprid and 20 ng/wasp sulfoxaflor in sucrose solution
1.0 or 10.0 µg/L imidacloprid in 250 mL water
25-140 ppb clothianidin or thiamethoxam treated candy (sugar and honey) *ad libitum*
1 or 10 µg/L imidacloprid in Biogluc® solution *ad libitum*
2uL of 5 or 500 ppb sulfoxaflor in sucrose solution, 4 times/day

0.004 or 4.375 ng thiamethoxam/µL in larval food
2.5, 5, 10, 20, 40 g clothianidin/ha solution evaporated onto a glass tube
2.061-11.350 mg/L acetamiprid evaporated as film onto a glass tube
0.5 µL of 8.18 or 16.84 ng acetamiprid/insect and 0 9.36 or 15.01 ng dinotefuran/insect
1 mL of 0.01, 0.1, 1, 10, 100, or 1000 µg/L evaporated as film onto a glass tube
0.5 or 1.0 mg/L imidacloprid solution
1 mL of 0.01, 0.1, 1, 10, 100, or 1000 µg/L evaporated as film onto a glass tube
0.45 kg imidacloprid/ha and 0.29 kg thiamethoxam/ha applied to soil sample
26.7 mg/L acetamiprid, 33.3 mg/L imidacloprid, nitenpyram,thiamethoxam, and 90.7 mg/L sulfoxaflor sp[illegible]
50 ppb imidacloprid and 4500 ppb thiacloprid in sucrose solution *ad libitum*

0.67 or 1.33 ng/larva/day clothianidin solution
0.15-6 ng/bee imidacloprid and 0.05-2 ng/bee clothianidin in sucrose solution
4.5 or 9 ppb clothianidin in sucrose solution *ad libitum*
4.5 ppm thiacloprid in sucrose solution *ad libitum*
1, 10, 100 nM or 1 µM imidacloprid and thiamethoxam in sucrose solution *ad libitum*
2 or 11 ppb thiamethoxam in sucrose solution *ad libitum*
5 ppb imidacloprid in sucrose solution *ad libitum*
20 uL/bee of 11.2, 22.5, 56.2, 112.4 ppb imidacloprid in sucrose solution
10 ppb thiamethoxam in sucrose solution *ad libitum*
1.05-6.3 ng/wasp acetamiprid, 5.25-21 ng/wasp flupyradifurone, 0.52-2.63 ng/wasp sulfoxaflor solution i[illegible]
0.4 ng/wasp imidacloprid solution in acetone
150 ng acetamiprid/bee
2 uL of nectar from plants treated with 5 mL of 1 mL/L imidacloprid solution
1-1000 nM neonicotinoid solution
50 ppb imidacloprid and 4.5 ppb thiacloprid solution *ad libitum*
2 µL/larva/day of 17.7 ppb imidacloprid in sucrose solution
10 µL of 8.9 or 88.7 ppb imidacloprid in sucrose solution
1.05-6.3 ng/wasp acetamiprid, 5.25-21 ng/wasp flupyradifurone, 0.52-5.25 ng/wasp sulfoxaflor solution i[illegible]
1.25 or 1.5 µg/mL imidacloprid in honey/water solution *ad libitum*
1.0 µg/mL imidacloprid in honey/water solution *ad libitum*
0.1% of 0.48 mg/L thiamethoxam solution sprayed in petri dishes
0.05, 0.18, or 0.48 mg/L thiamethoxam solution sprayed in petri dishes
0.1% of 0.98 mg/L thiamethoxam solution sprayed in petri dishes
0.13, 0.43, 0.98 mg/L thiamethoxam solution sprayed in petri dishes
0.45 kg imidacloprid/ha and 0.29 kg thiamethoxam/ha applied to soil sample
2.5, 5, 10, 20, 40 g clothianidin/ha solution evaporated onto a glass tube
5 µL of 0.11 mg/cm2 imidacloprid solution and 0.26 mg/cm2 thiamethoxam solution
10 µL of 0.01 mg/cm2 imidacloprid and thiamethoxam solution
Prey eggs and leaves dipped in 5 ppb imidacloprid solution and dried

Petioles of leaves with prey eggs were dipped in 0.78 mg/L thiamethoxam solution and dried
1081.1, 324.3, 108.1, 32.4, 10.8, and 1.1 ng thiamethoxam/g body weight in 20 uL food mixture
1 µL 0.003 or 0.037 mg/L acetamiprid solution
0.008-125 ng clothianidin/mg cat food, 0.008-8.3 ng imidacloprid/mg cat food, 0.0008-8.3 ng thiamethox
1 µL of 0-0.01 mg/cm2 imidacloprid solution and 0.04 mg/cm2 thiamethoxam solution
Leaves and prey eggs sprayed with highest label rate 144.0 mg thiacloprid/L
0.5 mL of 18.67 mg/L imidacloprid solution evaporated as film onto a glass tube
3.9 mg/cm^2 acetmiprid, 1.7 mg/cm^2 imidacloprid, 4.5 mg/cm^2 thiacloprid, or 3.7 mg/cm^2 thiametho
1183.5 ng/cm^2 imidacloprid solution sprayed on dorsal side of spider
1183.5 ng/cm^2 imidacloprid, 126 or 512.4 ng/cm^2 acetamiprid, 210 ng/cm^2 thiamethoxam, 472.7 or 7

4 ppb thiamethoxam and 1 ppb clothianidin in honey/pollen *ad libitum*
4 ppb thiamethoxam and 1 ppb clothianidin in honey/pollen *ad libitum*
0.01792 µg/cm^2 imidacloprid evaporated onto a glass vial
0.63-6.3 ng/wasp acetamiprid, 2.63-21 ng/wasp flupyradifurone, 2.63 ng/wasp sulfoxaflor solution in ace
0.1, 0.4, or 1.1 ng/wasp imidacloprid solution in acetone
6.32 or 19.9 µL/cm^2 neonicotinoid solutions applied to filter paper
4 ppb thiamethoxam and 1 ppb clothianidin in honey/pollen *ad libitum*
20 ng/g thiamethoxam in sucrose solution *ad libitum*
1.5, 4.5, or 10 ng/g thiamethoxam in sucrose solution *ad libitum*
0.7, 1.4, 6, 12 µg/kg imidacloprid in sucrose solution *ad libitum*
0.08-125 µg/L imidacloprid in sucrose solution *ad libitum*
1 or 4 ppb thiamethoxam in sucrose solution *ad libitum*
5 ppb sulfoxaflor in sucrose solution *ad libitum*
5 ppb imidacloprid in sucrose solution *ad libitum*
0.1 or 1 ng imidacloprid/bee
21.4%, 240 g/L imidacloprid soil spray application
2, 10, or 100 ppb imidacloprid in sucrose solution *ad libitum*
4.88 g/L thiacloprid solution sprayed onto aphid prey
10 mL of 4.5 or 30 µg/L thiamethoxam placed in nesting tubes
Seeds treated with 50 mg/100 kg thiamethoxam
0.0053, 0.053, or 0.53 mg thiamethoxam/L solution evaporated onto a glass tube
Undiluted pesticide containing imidacloprid applied to plates
2.5, 5, 10, 20, 40 g clothianidin/ha solution evaporated onto a glass tube
0.5-1 µL of 1-200 mg acetamiprid/L
1 µL 0.003 or 0.037 mg/L acetamiprid solution

0.3 g/L thiamethoxam or 0.75 mL/L immidacloprid solution as soil treatment or 0.1 g/L thiamethoxam or
0.032 g imidacloprid/200 mL solution applied to soil
0.56 kg imidacloprid/ha foliar spray or systemic fiedl treatment

0.56 kg imidacloprid/ha foliar spray or systemic fiedl treatment
seeds treated with 0.0756 or 0.152 mg/seed thiamethoxam

unds, and exposure doses.

Administration	Dosage Duration	Life Stage at exposure
Oral	24 hours	Adult
Oral	4 days	Adult
Oral	90 minutes	Adult
Oral	Once	Adult
Topical (Thorax)	Once	Adult
Oral	24 hours	Adult
Oral	10 days	Adult
Oral	Once-2 days	Adult
Oral	25 minutes	Adult
Oral	10 minutes	Adult
Oral	12 days	Adult
Oral	7 days	Adult
Oral	Once	Adult
Oral	1 hour	Adult
Residue	48 hours	Adult
Oral and Residue	1-30 days	Adult
Topical (Abdomen)	Once-48 hours	Adult
Oral	Daily for 4 days	Adult
Aquatic	9 days	Larva
Residue	throughout lifespan	Egg to Adult
Oral	1-8 days	Adult
Oral	24-72 hours	Adult
Oral	1-8 days	Adult
Oral	1-6 days	Adult
Aquatic	96 hours	Larva
Oral	Once	Adult
Oral	11-12 days	Adult
Oral	Daily for 6 days	Larva
Oral	Once	Adult
Oral	21, 24, or 45 days	Larva, Pupa, Adult
Oral	Once	Adult
Oral	Once	Adult
Aquatic	10-11 days	Larva
Oral	4-8 days	Adult
Oral	10 days	Adult
Oral	10 days	Larva

Oral	48 hours	Larva
Film Residue	Until adult emergence	Larva
Film Residue	12-24 hours	Larva
Topical (Abdomen)	Once	Larva
Film Residue	8 hours	Adult
Spray	Once	Adult
Film Residue	8 hours	Adult
Soil	2 days	Adult
Residue	24 hours	Adult
Oral	5 weeks	Adult

Oral	3 days	Larva
Oral	20 minutes	Adult
Oral	7-13 days	Adult
Oral	19-29 days	Adult
Oral	24 hours	Adult
Oral	10 days	Adult
Oral	17-37 days	Adult
Oral	Once	Adult
Oral	9-10 days	Adult
Topical (Abdomen)	Once	Adult
Topical (Abdomen)	Once	Adult
Oral	Once	Adult
Oral	Once	Adult
Dissected brain		Adult
Oral	6 weeks	Adult
Oral	Daily for 6 days	Larva
Oral	Once	Adult
Topical (Abdomen)	Once	Adult
Oral	3 weeks	Adult
Oral	2 weeks	Adult
Residue	12 hours	Adult
Residue	12 hours	Adult
Residue	12 hours	Adult
Residue	12 hours	Adult
Soil	2 days	Adult
Film Residue	Until adult emergence	Larva
Topical	Once	Adult
Topical	Once	Adult
Oral and Residue	24 hours	Adult

Oral and Residue	24 hours	Adult
Oral	Daily for 4 days	Adult
Topical	Once	Larva
Oral	24 hours	Adult
Topical	Once	Adult
Residue	24 hours	Nymph
Residue	Until adult emergence	Larva
Oral	30 minutes	
Topical	Once	
Topical	Once	

Oral	36 days	Adult
Oral	36 days	Adult
Residue	48 hours	Adult
Topical (Abdomen)	Once	Adult
Topical (Abdomen)	Once	Adult
Topical	1 hour	
Oral	36 days	Adult
Oral	12 days	Adult
Oral	4 days	Adult
Oral	2 weeks	Adult
Oral	13 days	Adult
Oral	14 days	
Oral	2 weeks	Adult
Oral	17-37 days	Adult
Oral	12 days	Adult
Residue	Once	Adult
Oral	48 hours	Adult
Fed and Residue	24 hours	Adult
Residue	64 weeks	Larva, Adult
Residue	Until pupation	Larva, Adult
Film Residue	Until adult emergence	Larva
Residue	throughout lifespan	Egg to Adult
Film Residue	Until adult emergence	Larva
Topical	Once	Larva
Topical	Once	Larva

Residue		
Residue		
Residue		

Residue		
Residue		
